# 

**Published:** 1875-09

**Authors:** 


### TABLE OF CONTENTS.

ORIGINAL COMMUNICATIONS.

On the Uses of the Binder in Parturiênt Women. By P. H. Clarke, M. D., Mason City, West Va.. .......... 513

Plaster Paris in the Treatment of Fractures. By E. H. Trickle, M. D., of Syracuse, Ohio ........ 521

"Rotheln." By J. C. Bishop, Middleport, Ohio.. ........ 526

An Easily Effectual Method of Artificial Respiration. By J. D. Mattison, Chester, N. J. ........ 529

Cases in Practice. By F. K. Bailey, M. D., Knoxville, Tenn........ 532

Mastitis Treated with Phytolacca Decandra. By T. Curtis Smith, M. D., Middleport, Ohio ........ 536

Abscess of Liver. By G. A. Baxter, M. D ........ 538

SELECTIONS.

Displacements of the Uterus. Medical and Surgical Society of Baltimore... 540

Operation for Varicocele by the Subcutaneous Wire-Loop........ 545

Two New Differential Signs in Dislocations of the Shoulder. By Prof. Frank H. Hamilton, M. D........ 547

The Mortality of Child-Bed ........ 549

EXTRACTS AND GLEANINGS.

Incontinence of Urine—554. Puerperal Fever—555. A New Instrument for Operating on Vesico-Vaginal Fistula—558. On Extraction of Cataract by a Median Section through the Cornea—559. An Improved Method of Treating Certain Cases of Cataract requiring Extraction—559. A New Method for Healing Ulcers—561. Diphtheria of a Vaccination Pustule followed by General Diphtheria—561. Diabetes—562. Heart Diseases—Treatment—562. Hygiene of Incurable Heart Disease—563. Some of the Therapeutic Properties of Jaborandi—563. A Case of Lympharrhagia—564. Indian Hemp in Flooding—564. Ointment for "Snuffles" in Infants—565. The Influence of the Use of Wind Instruments on Chest Affections—565. Diet of Infants affected with Acute Intestinal Catarrh—566. Glycerin in the Treatment of Diabetes—566. Poisoning by Strawberries—566. The Dyspepsia of Smokers—567. Local Treatment of Lichen Urticatus—567. Syphilitic Ghosts—567.

EDITORIAL AND MISCELLANEOUS.

Medical Societies and Medical Journals........ 568

Meetings of Medical Societies in Richmond........ 570

A Copartnership of Doctors, Homœopathy and Allopathy........ 571

Medical Advertising........ 572

Encouragement........ 574

Poisoning by Opium of a Suckling Babe through its Mother ........ 575

Treatment of Varicose Veins ........ 575

